# Research progress on the microbiota-gut-brain axis in autism spectrum disorder: a narrative review

**DOI:** 10.3389/fmicb.2026.1824546

**Published:** 2026-08-12

**Authors:** Songqiu Qin, Yuan Lin, Yimeng Chen, Sitong Yao, Dongsheng Zhou, Chong Zhang, Jianguo Yan, Yi He, Yali Zhou

**Affiliations:** 1 Institute of Pathogenic Biology, Guilin Medical University, Guilin, Guangxi, China; 2 Guangxi Medical and Health Key Cultivation Discipline Construction Project, Guilin, Guangxi, China; 3 Department of Infectious Diseases, The Second Affiliated Hospital of Guilin Medical University, Guilin, China; 4 Department of Neurology, The Second Affiliated Hospital of Guilin Medical University, Guilin, China; 5 Guangxi Key Laboratory of Brain and Cognitive Neuroscience, Guilin Medical University, Guilin, Guangxi, China

**Keywords:** autism spectrum disorder, gut microbiota, mechanism of action, microbiota-gut-brain axis, treatment strategies

## Abstract

Autism spectrum disorder (ASD) is a group of neurodevelopmental disorders characterized by deficits in social interaction and the presence of repetitive, stereotyped behaviors, often accompanied by gastrointestinal symptoms. The prevalence of ASD is on the rise, and the lack of specific pharmacological treatments places a significant burden on families and society. However, the discovery of the microbiota-gut-brain axis (MGBA) offers new opportunities for research into ASD. The gut microbiota, a core component of the MGBA, engages in bidirectional communication with the central nervous system through neural, immune, and metabolic pathways. Dysregulation of the gut microbiota is closely associated with the pathogenesis and progression of ASD. Children with ASD often exhibit specific alterations in their gut microbiota, including an increased abundance of Firmicutes and Proteobacteria, a decreased abundance of Bacteroidaceae, and abnormalities in short-chain fatty acid metabolism. These alterations are associated with neurotransmitter imbalances and impaired intestinal barrier function, which are thought to subsequently contribute to neuroinflammation. Given these insights, intervention strategies targeting the gut microbiota-such as probiotics/prebiotics supplementation, fecal microbiota transplantation (FMT), and gluten-free/casein-free diets-show promise in alleviating gastrointestinal symptoms and core behavioral deficits in children with ASD. This narrative review synthesizes the fundamentals of the MGBA, critically examines the mechanisms linking gut microbiota to ASD, discusses recent advances in related treatments and their methodological limitations, and aims to provide insights for future research and the potential development of precise, microbiota-based interventions for ASD.

## 1 Introduction

Autism spectrum disorder (ASD) is a group of developmental disorders that significantly impact children’s mental health. Key characteristics of ASD include persistent deficits in social communication and social interaction, which restrict and impair daily functioning and cannot be attributed to general developmental delays. Additionally, individuals with ASD often exhibit narrow, repetitive patterns of behavior, interests, or activities (Hirota and King, 2023). Cross-sectional epidemiological studies estimate the prevalence of ASD in China to be 0.70%, which translates to approximately 1 in 143 children. Notably, the prevalence is significantly higher in boys compared to girls (0.95% vs. 0.30%) (Zhou et al., 2020). Related research indicates that the prevalence of ASD has increased rapidly in recent years (Salari et al., 2022). However, the complex pathogenesis and lack of specific drug treatments lead to prolonged and costly therapy, placing significant physical and psychological pressure on the parents of affected children and resulting in a substantial societal burden.

Gut microbiota (GM) refers to the microorganisms residing in the gastrointestinal (GI) tract, which play a crucial role in the bidirectional communication between the gut and the brain, including the regulation of brain development and behavioral performance. Numerous studies have demonstrated that alterations in the gut microbiota are linked to various neurodevelopmental, neurodegenerative, and neuropsychiatric disorders, such as depression, schizophrenia, and autism spectrum disorder (Góralczyk-Bińkowska et al., 2022). Modifying the gut microbiota to alleviate autism-like behavioral symptoms in children with ASD presents a promising treatment approach.

As such, this narrative review provides a comprehensive synthesis and critical analysis of the current literature on the MGBA in ASD. Our aim is to integrate broad and heterogeneous evidence spanning mechanisms, microbiota profiles, and various interventions (probiotics, fecal microbiota transplantation, and diets). We explicitly define this work as a narrative review, distinct from a systematic review, and therefore it does not adhere to PRISMA guidelines. Instead of a formal quality appraisal, we have incorporated throughout the manuscript a critical discussion of methodological limitations, contradictory findings, and varying levels of evidence (detailed in section “4 Mechanisms of the microbiota-gut-brain axis in autism,” “5 Treatment strategies based on the microbiota-gut-brain axis”), to offer a balanced and rigorous perspective on the field.

## 2 Basic concepts of the microbiota-gut-brain axis

The microbiota-gut-brain axis (MGBA) has emerged as a focal point in medical research in recent years. This axis represents a bidirectional communication system that connects the brain and gastrointestinal microbiota through neural, metabolic, endocrine, and immune pathways (Ahmed et al., 2022). Many studies indicate that the gut microbiota plays a vital role in this complex interactive network (Liu et al., 2022). Gut microbes, primarily bacteria, create a vast microscopic ecosystem within the human gut, characterized by an exceptionally high microbial density and rich genetic diversity (Tian and Chen, 2024). Their numbers are 10 times the total number of human cells (Młynarska et al., 2022), and their total gene count is 150 times that of human genes. Thus, the gut microbiota plays a vital and complex role in influencing host health (Liu et al., 2023; Zhang et al., 2022). Gut microbes modulate brain development and function through multiple pathways, including tryptophan metabolism, the vagus nerve, the immune system, neurotransmitters activity, the hypothalamic-pituitary-adrenal (HPA) axis, and the production of microbial metabolites (Goyal et al., 2021). A stable gut microbiota is crucial for maintaining the balance between intestinal barrier integrity and inflammation. This balance is thought to positively influence brain function and modulate the host’s mood, cognition, and behavior, ultimately supporting overall health through the microbiota-gut-brain axis (Wang X. et al., 2023). Simultaneously, the brain is capable of maintaining the microecological balance of the gut, which in turn influences the structure and function of the gut microbiota, thereby affecting host health (Xue et al., 2023). A burgeoning body of research indicates that dysregulation of this axis may be associated with cognitive impairment and various mental health disorders, including Alzheimer’s disease, Parkinson’s disease, and ASD (Ma et al., 2024). This discovery offers new insights and directions for intervening in and treating these diseases by modulating the gut microbiota.

## 3 The relationship between autism and gut microbiota

### 3.1 Gut microbiota dysbiosis

A growing body of evidence suggests that children with ASD often exhibit alterations in their gut microbiota composition, a state commonly referred to as dysbiosis. However, it is critical to interpret these findings with caution. The observed microbial signatures are predominantly derived from cross-sectional, observational studies, which cannot establish causality. The heterogeneity among studies is substantial, and the reported changes are not always consistent. This variability likely reflects the complex interplay of factors such as subject-specific variables (genetics, age, diet, geographic location, GI comorbidities, and medication use) and technical discrepancies (sample collection, sequencing platforms, and bioinformatics pipelines). With these caveats in mind, we summarize the most commonly reported compositional trends at different taxonomic levels.

At the phylum level, Firmicutes, Bacteroidetes, Proteobacteria, and Actinobacteria dominate the gut ecosystems of both children with ASD and typically developing controls (Zou et al., 2020).

However, their relative abundances often differ, as summarized in Table 1.

**TABLE 1:** Taxonomic alterations of gut microbiota in children with autism spectrum disorder (ASD) compared to neurotypical controls.

Taxonomic level	Increased in ASD (consistent findings)	Decreased in ASD (consistent findings)	Conflicting results (direction varies by study)
Phylum	Firmicutes (Lewandowska-Pietruszka et al., 2023), Proteobacteria (Vernocchi et al., 2022), Actinobacteria (Xie et al., 2022)	/	Bacteroidetes [↑ in Iglesias-Vázquez et al. (2020); ↓ in Ha et al. (2021)]
Family	Veillonellaceae (Chang et al., 2024), Bifidobacteriaceae (Komijani et al., 2025), Enterobacteriaceae (Huang et al., 2021), Clostridiaceae (David et al., 2021), Pasteurellaceae (Vernocchi et al., 2022)	Bacteroidaceae (Andreo-Martínez et al., 2020; Zou et al., 2020), Lachnospiraceae (Chen et al., 2020)	/
Genus	*Sutterella*, *Lactobacillus*, *Klebsiella* (Vernocchi et al., 2022); *Parabacteroides*, *Phascolarctobacterium* (Yang C. et al., 2024); *Desulfovibrio*, *Alistipes* (Zang et al., 2023); *Megamonas* (Zou et al., 2020)	*Veillonella* (Strati et al., 2017); *Lachnospira* (Yang C. et al., 2024); *Flavonifractor*, *Dialister* (Zou et al., 2020); *Coprococcus* (Iglesias-Vázquez et al., 2020); *Citrobacter*, *Megasphaera*, *Escherichia*, *Shigella* (Ye et al., 2021); *Enterococcus* (Ahrens et al., 2024)	*Haemophilus* [↑ in Vernocchi et al. (2022); ↓ in Zou et al. (2020)], *Roseburia* [↑ in Vernocchi et al. (2022); ↓ in Niu et al. (2019)], *Clostridium* [↑ in Iglesias-Vázquez et al. (2020); ↓ in Zou et al. (2020)], *Ruminococcus* [↑ in Iglesias-Vázquez et al. (2020); ↓ in Niu et al. (2019)], *Bacteroides* [↑ in Zou et al. (2020); ↓ in Chen et al. (2020)], *Prevotella* [↑ in Zou et al. (2020); ↓ in Abuljadayel et al. (2024)], *Faecalibacterium* [↑ in Ye et al. (2021); ↓ in Ding et al. (2020)], *Akkermansia* [↑ in Zurita et al. (2020); ↓ in Ye et al. (2021)], *Blautia* [↑ in Lewandowska-Pietruszka et al. (2023); ↓ in Levkova et al. (2023)], *Streptococcus* [↑ in Wang H. et al. (2023); ↓ in Levkova et al. (2023)], *Bifidobacterium* [↑ in Dan et al. (2020); ↓ in Andreo-Martínez et al. (2022)]
Fungi	*Saccharomyces*, *Saccharomyces cerevisiae* (Zou et al., 2021); *Candida* (Zeng et al., 2025)	*Aspergillus versicolor*, *Aspergillus* (Zou et al., 2021)	/

Increased: ↑; Decreased: ↓

The contradictory findings regarding specific microbial taxa underscore a central challenge in ASD microbiome research. These discrepancies likely arise from a combination of factors, which we discuss in detail in section “4.1 Neural pathway” (Methodological Heterogeneity and Limitations). Briefly, key contributors include: (1) Biological and environmental heterogeneity among study populations (e.g., genetics, age, geography, habitual diet); (2) Unmeasured confounding variables prevalent in ASD, such as highly selective eating patterns, gastrointestinal comorbidities, and medication use (e.g., antibiotics, psychotropics); (3) The problem of reverse causality, whereby ASD-associated behaviors (e.g., restricted diets, altered gut motility) may themselves drive changes in microbiota composition; and (4) Technical variability in sample handling, sequencing methodologies, and bioinformatic analyses. These factors collectively limit the reproducibility and interpretability of cross-sectional comparisons.

### 3.2 Gastrointestinal symptoms

A growing body of research indicates that patients with ASD frequently experience gastrointestinal symptoms, including abdominal pain, diarrhea, constipation, and picky eating. A survey by Holingue et al. (2022) of 334 children with ASD found that constipation and flatulence were relatively common, followed by other abnormal gastrointestinal symptoms including diarrhea, alternating diarrhea and constipation, abdominal pain, and strong food preferences. Individuals with ASD exhibited GI symptoms more frequently than typically developing individuals without ASD symptoms (47.6% vs. 24.3%). Additionally, among those with ASD, girls reported a higher frequency of GI symptoms compared to boys (97.1% vs. 87.6%) (Babinska et al., 2020). Some studies point out that compared to autistic children without intestinal symptoms, the presence of intestinal comorbidities in autistic children may exacerbate the core symptoms of ASD (Yang T. et al., 2024). ASD patients with gastrointestinal symptoms exhibited higher levels of anxiety, increased physical discomfort, and reduced social interaction compared to those without gastrointestinal (Martínez-González and Andreo-Martínez, 2019). Studies have found that children with ASD who exhibit gastrointestinal symptoms have a higher detection rate of emotional problems and elevated behavioral problem scores compared to those without such symptoms (Babinska et al., 2020).

In recent years, research on ASD and gastrointestinal symptoms has significantly advanced. Sadik et al. (2022) discovered a link between parental, particularly maternal, diagnosis of inflammatory bowel disease (IBD) and childhood autism. If a mother has an inflammatory disease such as IBD during pregnancy, it may impact the fetal gut development and immune regulation through mechanisms that are not yet clearly defined. This exposure has been associated with an increased likelihood of gastrointestinal symptoms in the child later on. Kim et al. (2022) found that prenatal exposure to maternal inflammation increases susceptibility to bacteria-induced intestinal inflammation, suggesting that offspring with ASD are more prone to experiencing intestinal inflammation as they grow. Such inflammation-induced susceptibility changes have been linked to disruptions in the gut microbial community, which are thought to contribute to impaired intestinal function and an increased likelihood of gastrointestinal symptoms. Furthermore, the use of antibiotics during pregnancy has been associated with a heightened risk of ASD in offspring. Studies suggest that prenatal exposure to antibiotics can elevate the risk of ASD by 1.1 to 1.5 times (Love et al., 2024). Importantly, these prenatal exposures are thought to exert their effects, at least in part, through alterations in early-life gut microbial colonization. It is plausible that maternal inflammatory bowel disease and antibiotic treatment during pregnancy disrupt the vertical transmission of gut microbiota from mother to offspring, leading to aberrant microbial community assembly in the neonatal gut (Love et al., 2024; Sadik et al., 2022). This disruption in early microbial colonization patterns may subsequently impair intestinal barrier maturation and immune system development, thereby contributing to both gastrointestinal symptoms and ASD pathophysiology through the microbiota-gut-brain axis. Thus, these epidemiological risk factors may be mechanistically linked to ASD via microbiota-mediated pathways. Li H. et al. (2024) suggest that, compared to typically developing children, children with ASD have poorer diets characterized by fewer food varieties, higher levels of inadequate or imbalanced dietary intake, and more severe constipation along with total gastrointestinal symptoms. Additionally, research by Tomova et al. (2020) found that among children with ASD, “picky eaters” had more gastrointestinal diseases, with abdominal pain and constipation being significantly more frequent compared to “non-picky eaters.” Picky eating can result in excessive carbohydrate intake and insufficient fiber in the diet, which negatively affects intestinal digestion. This imbalance is frequently associated with constipation and may exacerbate the gastrointestinal symptoms commonly observed in children with ASD.

## 4 Mechanisms of the microbiota-gut-brain axis in autism

### 4.1 Neural pathway

The vagus nerve is a mixed nerve, consisting of approximately 80% afferent fibers and 20% efferent fibers. As the longest, most complex, and most widely distributed of the 12 pairs of cranial nerves, it plays a crucial role in the parasympathetic nervous system (Mandalaneni and Rayi, 2023). The gut-brain axis involves complex interactions among several components: the gut microbiome, gut endocrine cells, the mucosal immune system, the enteric nervous system, the autonomic nervous system, and central processes that receive vagal input (Hokanson et al., 2024; Kong et al., 2021). As the main pathway for information exchange between the gastrointestinal tract and the brain, the vagus nerve extensively regulates various bodily functions (Zhang et al., 2020) and plays a significant role in the microbiota-gut-brain axis (Powley, 2021). Studies have shown that the vagus nerve is capable of perceiving various types of intestinal information, including mechanical, chemical, and hormonal signals (Lai et al., 2024; Lowenstein et al., 2023; McDougle et al., 2024), thereby influencing the activity of the central nervous system. Numerous studies indicate that activation of the vagus nerve sends signals through afferent nerve fibers to the nucleus tractus solitarius (NTS; Kong et al., 2021), then relayed through the parabrachial nucleus (PB), ascending to the locus coeruleus (LC) and dorsal raphe nucleus (DRN; Andalib et al., 2023; Liu T. T. et al., 2024), and subsequently projecting to different brain regions such as the hypothalamus, amygdala, and cerebral cortex (Agetsuma et al., 2025), regulating brain activity and being crucial for emotional regulation in children with ASD (Sun et al., 2024). The gut microbiota produces a range of neurotransmitters and metabolites, including dopamine (DA), γ-aminobutyric acid (GABA), 5-Hydroxytryptamine (5-HT), short-chain fatty acids (SCFAs), and phenolic compounds. These substances are thought to influence the central nervous system through the vagus nerve, eliciting various responses and potentially contributing to the development of neurodegenerative diseases (Bhatia et al., 2023; Yaghoubfar et al., 2020). Liu et al. (2021) administered *Lactobacillus rhamnosus* to vagotomized mice and observed a reduction in anxiety-like behavior. This finding provides evidence that the vagus nerve is part of the bidirectional communication pathway between gut microbes and the brain (Liu et al., 2021). Similarly, Sgritta et al. (2019) found that *Lactobacillus reuteri* improved social behavior in ASD mouse models via the vagal pathway, suggesting that the vagus nerve represents an important communication channel between the gut and the brain. Zou et al. (2024) discovered that *Salmonella* may activate the host’s central nervous system (CNS) via the vagus nerve, and this activation has been associated with anxiety-like behavior and local inflammation in the CNS. Their findings present a novel and effective approach for targeting the gut-brain axis to prevent related behavioral abnormalities (Zou et al., 2024).

### 4.2 Immune pathway

The gut is not only a site for microbial colonization; it also plays a crucial role as an organ in the human body that performs immune functions (Wang et al., 2024). The immune system is essential for the bidirectional regulation between the microbiota, gut, and brain, allowing the gut and brain to influence each other (Alharthi et al., 2022). The gut microbiota not only influences the development and maturation of the immune system and its cells (Donald and Finlay, 2023), but can also regulate the intestinal mucosal immune system, the systemic immune system, and the function of central nervous system immune cells, thereby directly or indirectly regulating neural activity (Zhou et al., 2025).

The gut microbiota plays a crucial role in regulating the development, maturation, and function of immunity in both the local intestinal system and the central nervous system. This has been confirmed through experiments involving germ-free (GF) mice, which display an underdeveloped mucosal immune system. Notably, GF mice show reduced IgA secretion, smaller Peyer’s patches, and abnormal expression of Toll-like receptors (TLRs; Marietta et al., 2018). Additionally, GF mice exhibit impaired maturation and functional defects in brain microglia, along with weakened innate immune responses. These issues can be restored through colonization with a complex microbiota or by intervention with microbial-derived metabolites (Erny and Prinz, 2020; Erny et al., 2015). As the immune cells of the brain, abnormal activation and dysfunction of microglia are closely related to ASD (Luo and Wang, 2024). Dysbiosis of the gut microbiota in ASD patients has been associated with intestinal barrier damage and increased intestinal permeability (Al-Ayadhi et al., 2021; Fasano, 2020; Liu S. et al., 2024). It is hypothesized that this may permit bacteria, toxins, and metabolites to translocate across the intestinal epithelium, potentially activating the immune system and contributing to a peripheral inflammatory state. In turn, this systemic inflammation may compromise the integrity of the blood-brain barrier, facilitating the entry of cytokines and microbial-derived substances into the brain. This intrusion may activate microglia and trigger neuroinflammation, which in turn has been implicated in various neurological symptoms (Davoli-Ferreira et al., 2021; Yadav et al., 2025; Zhou et al., 2025). Li et al. (2023) demonstrated evidence of intestinal barrier dysfunction and increased permeability in an autism mouse model, and postmortem studies have shown widespread microglial activation and chronic neuroinflammatory state in the brain tissue of ASD patients (Liao et al., 2020a,b). Secondly, a considerable proportion of ASD patients experience persistent immune dysfunction and chronic inflammatory responses. One study indicated differences in immune cytokine profiles between ASD patients and healthy controls, showing significantly elevated levels of various cytokines in plasma, including IL-2, IL-4, IL-6, TNF-α, and IFN-γ. Additionally, changes in specific bacterial taxa were found to correlate with the levels of peripheral blood cytokines in ASD patients. For instance, plasma IFN-γ levels were positively correlated with the relative abundance of *Pseudomonas*, *Streptomyces*, and *Clostridium*, while showing a negative correlation with the relative abundance of *Blautia* (Cao et al., 2021). Additionally, the gut microbiota can regulate T lymphocytes, which in turn shape and influence behavioral outcomes in ASD models. Park et al. (2025) discovered that GF BTBR mice exhibited lower percentages of brain-resident CD4+ and CD8+ T cells compared to SPF BTBR mice, along with an increased frequency of Tregs. Furthermore, the depletion of CD4+ T cells in SPF BTBR mice impacted the microglial phenotype, leading to a reduction in inflammatory M1 microglia and promoting a shift toward anti-inflammatory and tissue-repair-associated M2 microglia (Park et al., 2025). The Maternal Immune Activation (MIA) model is widely utilized to investigate mechanisms related to ASD. Importantly, the maternal gut microbiota may play a role in MIA, subsequently influencing fetal brain development (Mavel et al., 2025). Kim et al. (2017) demonstrated that pregnant mice colonized with the mouse commensal segmented filamentous bacterium (SFB) or human commensal bacteria, which induce intestinal Th17 cells, had a higher likelihood of producing offspring with MIA-associated abnormalities. Dendritic cells (DCs) from pregnant women exposed to MIA secreted IL-1β/IL-6/IL-23 and stimulated Th17 cells to produce IL-17a (Kim et al., 2017), thereby potentially increasing the risk of neurodevelopmental disorders in offspring, which may manifest as ASD-like behavioral phenotypes.

### 4.3 Metabolic pathway

Intestinal microbiota play a crucial role in the degradation and metabolism of food within the body. The metabolites they produce not only supply nutrients for their own growth but also help maintain and regulate the intestinal environment. Additionally, these metabolites facilitate bidirectional signaling both locally in the gut and peripherally. The gut microbiota is vital for normal brain development and behavior, with its metabolites significantly influencing various brain functions. These functions include essential processes such as neurodevelopment, neurotransmission, blood-brain barrier integrity, and neuroinflammation (Ahmed et al., 2022). When gut microbiota dysbiosis occurs, it is associated with abnormal digestive processes and metabolic disorders. During this time, substances that enter the central nervous system may adversely affect it, potentially contributing to mood and behavioral abnormalities. For example, early-life gut microbiota dysbiosis and the associated changes in metabolites, such as elevated p-cresol, can impair hippocampal development and neuroplasticity. This remodeling of the serum metabolome ultimately may contribute to hippocampal dysfunction, behavioral disorders, and ASD-like behaviors (Liu et al., 2022).

Gut microbes produce a variety of metabolites, primarily consisting of short-chain fatty acids, secondary bile acids, essential vitamins, and amino acids (Ullah et al., 2023), as well as various neuroactive substances and their precursors, such as dopamine, norepinephrine, and GABA. These substances can exert local effects in the gut or indirectly influence central functions.

5-Hydroxytryptamine (5-HT), an important neurotransmitter in the brain, stabilizes signal processing in the frontal lobe and regulates behavioral and emotional responses. While the brain synthesizes only a small portion of the body’s total 5-HT (less than 10%), over 90% is produced and secreted by enterochromaffin cells in the gut, utilizing tryptophan under the regulatory influence of the gut microbiota (Ahmed et al., 2022). Microbial metabolites, such as tyramine and deoxycholic acid, can stimulate the synthesis of 5-HT, while norepinephrine and indole can promote its release (Ahmed et al., 2022). Beyond the 5-HT pathway, tryptophan is also metabolized via the kynurenine pathway. In the context of ASD, pro-inflammatory cytokines can activate indoleamine 2,3-dioxygenase (IDO), shifting tryptophan metabolism toward kynurenine production at the expense of serotonin synthesis. This generates neuroactive metabolites such as quinolinic acid (neurotoxic) and kynurenic acid (neuroprotective), and an altered quinolinic acid/kynurenic acid balance may contribute to neuroinflammation and excitotoxicity relevant to ASD neuropathology. Thus, the gut microbiota crucially orchestrates the balance between these two tryptophan metabolic routes, with direct implications for ASD pathophysiology.

Gut bacteria convert primary bile acids into secondary bile acids, which act as signaling molecules via receptors (FXR, TGR5) expressed in the gut, liver, and brain. Altered bile acid profiles have been reported in children with ASD and may influence intestinal barrier function and immune regulation, though the functional significance of these changes remains to be established.

p-Cresol, a bacterial fermentation product of tyrosine, has been detected at elevated levels in children with ASD and may impair mitochondrial function and neuronal activity. Lipopolysaccharides (LPS), components of Gram-negative bacterial membranes, can enter the circulation when intestinal permeability is compromised and are potent inducers of systemic inflammation linked to neuroinflammation.

Collectively, these emerging findings underscore the need for comprehensive metabolomic approaches in ASD research–moving beyond SCFAs alone to capture the full complexity of microbial metabolite-host interactions. Elucidating the functional contributions of these diverse metabolites to the MGBA in ASD remains an important priority for future investigation.

SCFAs are monocarboxylic acids with fewer than 6 carbon atoms, primarily including acetate (AA), propionate (PPA), and butyrate (BA), among others. Results from multiple studies indicate increased SCFAs in the feces of subjects with autism spectrum disorder, with a notable characteristic being elevated propionate levels (Lagod and Naser, 2023). SCFAs are the primary metabolic products generated by the anaerobic fermentation of undigested carbohydrates, including oligosaccharides, non-starch polysaccharides, and resistant starch. These compounds play a crucial role in gut health and function. SCFAs are well-established as an energy source for intestinal epithelial cells (IECs) and are known to regulate IEC proliferation, differentiation, and the function of specific subpopulations, such as enteroendocrine cells, through various mechanisms. Additionally, SCFAs have been shown to influence intestinal motility, promote barrier function, support host metabolism, and inhibit the growth of harmful bacteria (Martin-Gallausiaux et al., 2021). Simultaneously, SCFAs have been shown to modulate the immune system, epigenetics, and neuroplasticity in the central nervous system; the brain, energy balance, and metabolism are all affected by them (Ullah et al., 2023). SCFAs possess neuroactive properties that impact the central nervous system. When taken up by the CNS, SCFAs promote microglial maturation, affect neural signaling, regulate neurotransmitter and neurotrophic factor levels, and influence neural signal conduction. These effects ultimately contribute to CNS development (Settanni et al., 2021). Furthermore, SCFAs can exert broad effects on host metabolism, differentiation, and proliferation through gene regulation. Multiple studies indicate that 5%–20% of human gene expression is regulated by butyrate (Martin-Gallausiaux et al., 2021).

Among intestinal SCFAs, acetate levels are significantly higher than those of other SCFAs. This is largely due to the metabolic advantages of acetate-producing bacteria and their central role in microbial ecology. Acetate is primarily produced by anaerobic bacteria, including *Akkermansia*, *Bifidobacterium*, and *Bacteroides*, which ferment dietary fiber. Research indicates that in germ-free mouse models, exogenous acetate can promote microglial maturation and help regulate brain metabolic homeostasis (Hays et al., 2024). Propionate can be produced by various gut microbial metabolisms and has the ability to cross the blood-brain barrier, potentially inducing ASD-like behaviors. In comparison to neurotypical (ND) controls, individuals in the ASD group exhibited a significant increase in PPA-producing bacteria, including *Bacteroides* and *Desulfovibrio*. Additionally, related animal studies suggest that the direct administration of PPA can result in ASD-like symptoms and lead to alterations in ASD-related molecules in the brain (Lagod and Naser, 2023). Butyrate is the primary energy source for intestinal epithelial cells. It inhibits the release of pro-inflammatory cytokines and plays a crucial role in intestinal immune homeostasis. Related studies suggest it can improve repetitive behaviors in BTBR mice, indicating positive benefits for ASD (Tao et al., 2025). Butyrate can regulate gene transcription in the prefrontal cortex and enhance hippocampal dendritic spine plasticity in ASD mouse models, leading to improvements in social deficits and spatial memory function. In summary, dysbiosis of gut microbiota in children with ASD is significantly linked to disruptions in SCFA metabolism, which may worsen the core behavioral symptoms of ASD.

**FIGURE 1:**
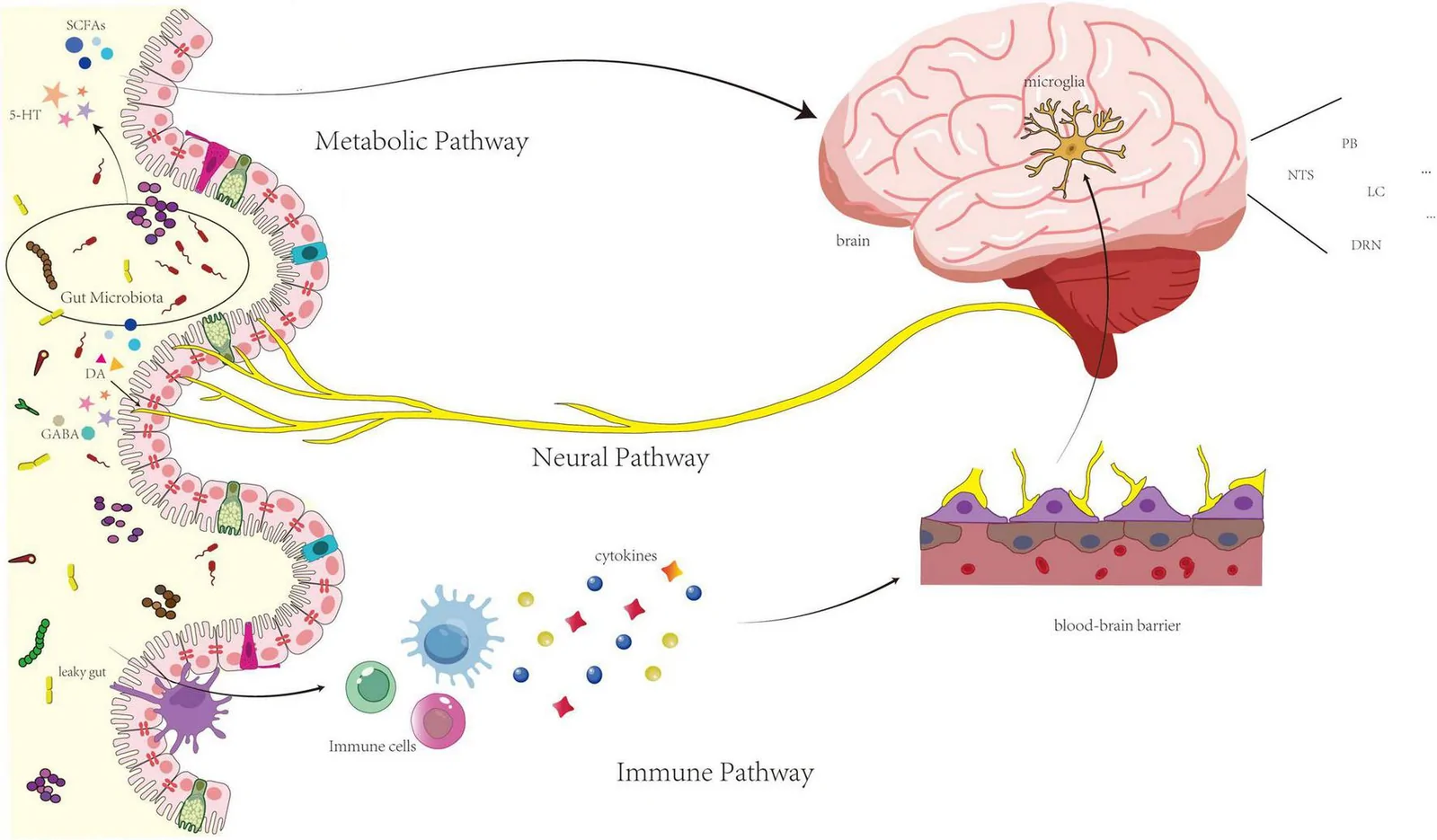
Schematic diagram of the microbiota-gut-brain axis. The regulatory mechanisms of the microbial gut-brain axis are mediated through three interconnected pathways: the vagal, immune, and metabolic pathways. The immune pathway comprises key components such as immune cells and cytokines. The microbial metabolite pathway involves substances like short-chain fatty acids (SCFA), 5-Hydroxytryptamine (5-HT), and γ-aminobutyric acid (GABA). The vagal pathway includes essential elements such as microglia, the nucleus tractus solitarius (NTS), the parabrachial nucleus (PB), the locus coeruleus (LC), the dorsal raphe nucleus (DRN), and dopamine (DA). These pathways function together to facilitate bidirectional communication between the central nervous system and the gut microbiota.

The three interconnected pathways described above–neural, immune, and metabolic– collectively constitute the complex regulatory network of the microbiota-gut-brain axis in autism spectrum disorder (ASD). A schematic overview of these pathways and their key components is presented in Figure 1, illustrating how gut microbiota-derived signals can influence brain function and behavior through multiple mechanistic routes.

These mechanistic insights–spanning neural, immune, and metabolic pathways–provide the biological rationale for the microbiota-targeted intervention strategies discussed in the following section. Each therapeutic approach, including probiotics/prebiotics, fecal microbiota transplantation, and dietary modifications, aims to modulate one or more of these interconnected pathways to restore gut homeostasis and potentially ameliorate both gastrointestinal and core behavioral symptoms in children with ASD. As we will discuss, however, the strength of evidence supporting each strategy varies considerably, and significant methodological challenges must be addressed before these interventions can be translated into routine clinical practice.

### 4.4 The top-down axis: brain-to-gut signaling

While the preceding sections have focused on bottom-up signaling (microbiota→brain), it is equally important to acknowledge the top-down direction (brain→microbiota) to fully appreciate the bidirectional nature of the MGBA. The hypothalamic-pituitary-adrenal (HPA) axis serves as a primary conduit for this top-down regulation. Psychological stress, a common comorbidity in ASD, activates the HPA axis, leading to the release of cortisol (in humans) or corticosterone (in rodents). Elevated glucocorticoid levels can directly alter intestinal barrier permeability, modulate gut motility, and influence the composition and function of the gut microbiota by affecting the intestinal luminal environment (Ahmed et al., 2022; Horn et al., 2022). For instance, chronic stress has been shown to reduce the abundance of beneficial genera such as *Lactobacillus* and *Bifidobacterium* while promoting the growth of potentially pathogenic bacteria (Horn et al., 2022). Furthermore, stress-induced alterations in autonomic nervous system output can indirectly shape the gut microbial ecosystem through changes in intestinal transit time, mucus secretion, and local immune responses. This top-down regulation is particularly relevant to ASD, as individuals with ASD frequently experience elevated levels of anxiety and stress, which may in turn exacerbate gut dysbiosis and gastrointestinal symptoms, creating a self-reinforcing cycle. Future studies should aim to delineate the relative contributions of bottom-up versus top-down signaling in ASD pathophysiology, as this distinction has important implications for the design of targeted interventions.

## 5 Treatment strategies based on the microbiota-gut-brain axis

### 5.1 Probiotics and prebiotics

Recent studies have shown a significant association between autism spectrum disorder (ASD) and gut microbiota dysbiosis (Li et al., 2017), with significant differences in gut microbiota between ASD patients and healthy controls (Wang Q. et al., 2023). Characteristics may include reduced abundance of beneficial bacteria such as *Bifidobacterium* and *Akkermansia* (Coretti et al., 2018; Pellegrino et al., 2023), and overgrowth of opportunistic pathogens such as *Clostridium* and *Desulfovibrio* (Morton et al., 2023; Wan et al., 2022). This imbalance is thought to impact the central nervous system through multiple mechanisms: Firstly, the abnormal synthesis of microbial metabolites, such as SCFAs (e.g., decreased butyrate and increased propionate), may compromise the integrity of the blood-brain barrier. Additionally, it can disrupt microglial maturation and interfere with synaptic pruning processes (Fock and Parnova, 2023). Secondly, microbiota disruption may contribute to abnormal synthesis of neurotransmitters such as GABA and 5-HT (Bravo et al., 2011). The imbalance in the GABA/glutamate ratio in the gut and brain of ASD patients is significantly correlated with social impairment. Additionally, elevated peripheral 5-HT levels may inhibit prefrontal cortex function through vagal transmission (El-Ansary and Al-Ayadhi, 2014; Kuo and Liu, 2022). Additionally, increased intestinal permeability may facilitate the translocation of lipopolysaccharides (LPS), which can activate systemic inflammatory responses (Heisler and O’Connor, 2015). Pro-inflammatory cytokines, such as IL-6 and TNF-α, have been implicated in worsening the tryptophan-kynurenine metabolic imbalance through upregulation of the IDO enzyme, potentially leading to the accumulation of neurotoxic metabolites (Tanaka et al., 2021). Targeting the mechanisms described above, probiotic and prebiotic intervention strategies demonstrate potential for precise regulation. Probiotics, which are live microbial preparations, can operate through three pathways: (1) Ecological reconstruction, such as *Bacteroides fragilis* inhibiting the growth of *Clostridium* by secreting antibacterial peptides (Deng et al., 2018); (2) Neurotransmitter regulation, e.g., increased *Bifidobacterium* can raise serotonin levels, improving ASD-related and gastrointestinal symptoms (Wang et al., 2020); (3) Immune modulation, e.g., probiotic supplementation can significantly alter social and emotional behaviors in rats and the levels of cytokines such as IL-6, IL-17a, and IL-10 in the blood (Adıgüzel et al., 2022; Wang et al., 2019).

It is crucial to emphasize, however, that probiotic effects are highly strain-specific; the clinical outcomes discussed hereafter are tied to the particular strains, doses, and formulations used in the cited studies. Despite promising findings, current research has notable limitations. First, the effects of specific strains are not fully elucidated. For instance, only certain strains of *L. reuteri* have been shown to improve social function (Mazzone et al., 2024). Moreover, probiotic formulation significantly affects efficacy; in a comparative study, significant improvement in overall ASD symptoms was found only in the multi-strain probiotic group, not the single-strain group (Lee et al., 2024). Second, the overall evidence base remains insufficient to fully clarify the potential of probiotics on ASD symptoms, and most existing clinical studies have focused on children, leaving a critical gap regarding intervention effects in adults with ASD (He et al., 2023). Despite these limitations, breakthroughs in synthetic biology and microbiome technology are paving the way for next-generation probiotic preparations. These innovations aim to achieve precise regulation by integrating the ‘strain-metabolite-neural circuit,’ thereby creating new models for ASD treatment.

To critically appraise the current evidence base, it is essential to stratify the available clinical data by methodological rigor. Among the clinical studies summarized in Table 2, only two are randomized, double-blind, placebo-controlled trials (RCTs; Liu et al., 2019; Mazzone et al., 2024), both of which employed modest sample sizes. Mazzone et al. (2024) reported that *Lactobacillus reuteri* supplementation improved social function in children with ASD, but found no significant effect on overall ASD severity. Similarly, Liu et al. (2019) demonstrated that *Lactobacillus plantarum* PS128 improved oppositional behaviors in a Taiwanese pediatric cohort. The remaining clinical evidence derives from open-label studies (Li N. et al., 2021) or preclinical animal models (Adıgüzel et al., 2022; Sgritta et al., 2019; Wang et al., 2019), which, while providing valuable mechanistic proof-of-concept, are inherently subject to significant bias, particularly placebo effects and lack of blinding. Multi-strain probiotic formulations combined with behavioral therapy have shown promise in reducing ATEC scores in one RCT (Li et al., 2021), yet the generalizability of these findings is limited by the small number of participants and the single-center design. Overall, while the preliminary evidence is encouraging, the field urgently requires larger, multi-center, double-blind, placebo-controlled RCTs with standardized outcome measures to establish the efficacy of probiotic interventions for ASD.

**TABLE 2:** Mechanisms of probiotics/prebiotics in modulating ASD symptoms: supporting evidence by study design.

Intervention (probiotics/prebiotics)	Mechanistic evidence	Clinical / behavioral evidence	Representative references
Long *Bifidobacterium*, *Lactobacillus acidophilus*, and *Enterococcus faecium* three-strain probiotic powder	Increase the level of the short-chain fatty acid butyrate; ↑ beneficial microbiota; ↓ harmful microbiota	RCT: multi-strain probiotics + ABA therapy reduced ATEC scores, improved gut dysbiosis	Li et al., 2021
Fecal microbiota transplantation (FMT)	Restores the gut microbiota, Facilitates the colonization of donor microbes	Open-label study: improved GI symptoms and some ASD behaviors	Li N. et al., 2021
*Lactobacillus reuteri*	Acts on the vagus nerve; ↑ oxytocin release	Pilot RCT: improved social function in children, but no effect on overall ASD severity	Mazzone et al., 2024; Sgritta et al., 2019
*Lactobacillus plantarum* PS128	Regulate brain neurotransmitter levels	A randomized, double-blind, placebo-controlled trial: improved oppositional behaviors in children with ASD	Liu et al., 2019
Prebiotics (tibetan tea polysaccharides, almond AP-1, B-GOS)	↑*Bifidobacterium*/*Lactobacillus*, ↑pro-inflammatory bacterial taxa; reduces LPS-induced inflammation	Combination of gluten-free, casein-free diet and B-GOS improves social behavior in children with ASD	Duque et al., 2021; Peng et al., 2024; Tan et al., 2023

RCT, randomized controlled trial; ABA, applied behavior analysis; ATEC, Autism Treatment Evaluation Checklist; GI, gastrointestinal; ASD, autism spectrum disorder; B- GOS, *Bifidobacterium longum*, galacto-oligosaccharides; ↑, increase; ↓, decrease.

Clinical studies indicate that microbiota interventions can significantly enhance core ASD symptoms. Table 2 presents the mechanisms through which probiotics and prebiotics modulate ASD symptoms via the gut-brain axis, along with the clinical evidence supporting these interventions.

The clinical evidence supporting probiotic interventions for ASD can be stratified by study design, as summarized in Table 2. Preclinical animal models have provided mechanistic proof-of-concept, demonstrating that specific strains (e.g., *Lactobacillus reuteri*, *Lactobacillus plantarum* PS128) can improve social behavior and modulate neurotransmitter levels via vagal and oxytocinergic pathways (Liu et al., 2019; Mazzone et al., 2024; Sgritta et al., 2019). Open-label and pilot clinical trials have reported encouraging signals, including improvements in gastrointestinal symptoms and select behavioral measures, though these findings must be interpreted cautiously given the absence of blinding and placebo controls (Li N. et al., 2021). Randomized controlled trials (RCTs) remain limited in number; available evidence from a small number of RCTs suggests that multi-strain probiotic formulations combined with behavioral therapy may reduce ATEC scores and improve gut dysbiosis (Li et al., 2021), while single-strain preparations have shown more modest and strain-specific effects on discrete behavioral domains (Liu et al., 2019; Mazzone et al., 2024). The overall evidence base, while promising, is constrained by small sample sizes, heterogeneity in outcome measures, and the inherent strain-specificity of probiotic effects.

### 5.2 Fecal microbiota transplantation (FMT)

Fecal Microbiota Transplantation (FMT) is an increasingly popular fecal bacterial therapy (Antushevich, 2020). FMT involves transplanting fecal gut microbiota from a healthy donor into the patient’s gastrointestinal tract. This process aims to restore normal microbial proportions and rebuild gut microbiota homeostasis, ultimately treating various diseases associated with gut dysbiosis (Green et al., 2020).

Fecal microbiota transplantation is used to treat various diseases, and the process typically involves several key steps: donor screening, preparation of donor fecal material, pre-procedure preparation for the patient, transplantation via an appropriate route, and post-transplant patient management with regular follow-up.

Donor screening is highly rigorous. To ensure the safety and reliability of the graft, a comprehensive series of health screenings must be conducted on the donors (He et al., 2021). According to the European Consensus Conference on Clinical Practice of Fecal Microbiota Transplantation, the first step for donors should be to accept a written questionnaire (Cammarota et al., 2017) to check whether the donors have infection risks (such as AIDS, syphilis, hepatitis C, etc., as well as exposure risks such as high - risk sexual behavior), and they should not suffer from metabolic syndrome, immune diseases, specific diseases, etc., nor use drugs that affect the intestinal flora, nor have the phenomena of smoking, excessive obesity, malnutrition, etc. Various indicators of the donor’s blood and feces need to be laboratory-tested to ensure complete health. In recent years, the question of whether it is preferable to use relatives as donors, unlike in organ transplantation, has become a subject of debate (Mullish et al., 2023). Some scholars have also suggested that gender differences should be considered for FMT (Vemuri et al., 2019). Some institutions have recruited volunteers from blood donation drives to serve as stool donors, which has helped reduce both recruitment costs and time (Jørgensen et al., 2017). With deepening research, many established stool banks are now available (Bottino et al., 2024).

The preparation process for fecal material may vary slightly between different institutions. Typically, it involves the following steps: first, take 50–60 g of fresh stool and dilute it with 200–300 mL of sterile saline. Next, mix the solution evenly and filter it to remove large particulate matter. After filtering, centrifuge the mixture to eliminate small impurities, then retain the supernatant for use. Finally, portion the supernatant for patient use (Collins and DeWitt, 2020; Varga et al., 2021). When obtaining donor stool from a stool bank, an additional thawing step is required as the stool is stored frozen (Keller et al., 2021).

Prior to FMT, depending on requirements, patients may need antibiotic pretreatment or bowel cleansing (Keller et al., 2021) to create conditions for transplantation. Some scholars recommend conducting a straightforward pre-FMT screening of the recipient to assess whether any adverse events following the FMT are attributable to the transplanted fecal material (Vindigni and Surawicz, 2017).

Fecal microbiota transplantation can be administered via various routes, mainly divided into upper and lower gastrointestinal tract delivery (Mullish et al., 2023); mid-gut delivery is rarely used clinically. Upper GI routes can use nasogastric or nasoduodenal tubes, or oral capsules (Allegretti et al., 2020). Lower GI routes deliver the preparation to the colon via colonoscopy or enema (Mullish et al., 2023). During the procedure, the prepared fecal microbiota suspension is infused into the patient’s gastrointestinal tract. Current clinical applications indicate that various infusion routes each have their own advantages and disadvantages. Therefore, the optimal approach should be chosen based on the patient’s specific condition to minimize operational risks (Sun et al., 2023). Anti-diarrheal medication is sometimes administered post-procedure to promote successful colonization. Studies indicate that minor adverse events can occur after FMT, primarily gastrointestinal issues, including diarrhea, abdominal pain, bloating, and constipation (Allegretti et al., 2020; Huang et al., 2022), which are natural microbial implantation reactions that usually resolve quickly and have little impact on the patient. Therefore, post-transplant management is essential to prevent unexpected complications. Additionally, regular follow-up (Sun et al., 2023) is essential to check patient outcomes and monitor transplant efficacy.

Currently, FMT is primarily used for *Clostridium difficile* infection (CDI; Popa et al., 2021), Irritable Bowel Syndrome (IBS; Wang M. et al., 2023), and inflammatory bowel disease (IBD), including Crohn’s Disease (CD) and Ulcerative Colitis (UC; Caldeira et al., 2020), with relatively promising cure rates. As research deepens, FMT is being applied to other extra-intestinal diseases associated with gastrointestinal dysfunction (Benítez-Páez et al., 2022), including ASD, with some positive results.

Numerous FMT animal experiments have demonstrated that injecting fecal suspension from ASD patients into experimental mice can induce symptoms similar to autism. In contrast, transplanting normal fecal microbiota has been shown to improve autistic-like behaviors in ASD model mice.

Sharon et al. (2019) conducted a study in which they transplanted gut microbiota from donors with ASD and typically developing (TD) individuals into germ-free (GF) mice. They discovered that colonization with ASD microbiota led to changes in the splicing of ASD-related genes in the mouse brain. Additionally, predictions derived from the microbiota and metabolome profiles of humanized microbiota mice indicated that specific bacterial taxa and their metabolites could modulate ASD behaviors. Furthermore, treating ASD mouse models with certain microbial metabolites resulted in improvements in behavioral abnormalities and modulation of neuronal excitability in the brain. Similarly, Xiao et al. (2021) transplanted fecal samples from TD and children with ASD into GF mice. They found that the fecal microbiome from children with ASD resulted in ASD-like behaviors, such as increased repetitive behaviors and reduced sociability. Additionally, this transplantation led to distinct microbial community structures and altered tryptophan and serotonin metabolism in the GF mice. Zheng et al. (2024) performed FMT on BTBR mice using human fecal microbiota. Their study demonstrated that humanized FMT selectively reversed ASD-like social deficit symptoms in these mice. This intervention had a positive impact by reducing toxins and increasing metabolites that are beneficial for mitochondrial and neurological health. The team of Wang J (Wang J. et al., 2023) transferred gut microbiota from healthy donors into a valproic acid (VPA)-induced autism mouse model, which alleviated the core symptoms of autism spectrum disorder by influencing serotonergic and glutamatergic synapse pathway signaling.

In daily life, emotional fluctuations significantly impact the gastrointestinal tract. Many patients with poor GI function experience discomfort, such as bloating, during periods of emotional distress. This phenomenon is linked to the gut microecosystem, highlighting the importance of gastrointestinal microbiota in disease progression. Given the positive preclinical results, researchers are increasingly focusing on FMT as a treatment for ASD patients. In the clinical domain, the available human evidence derives primarily from small, open-label studies and case series, with no published randomized, double-blind, placebo-controlled trials to date. A notable case is a small open-label study by Kang DW’s team (Kang et al., 2017). Initially, 18 ASD patients underwent 2 weeks of antibiotic treatment and bowel cleansing, followed by 7–8 weeks of FMT. At the end of the treatment, GI symptoms were reduced by approximately 80%, with significant improvements observed in constipation, diarrhea, indigestion, and abdominal pain. These improvements persisted for 8 weeks. Similarly, clinical assessments indicated a significant reduction in autism symptoms, which were also maintained for 8 weeks after treatment concluded. Deep sequencing analysis of bacteria and phages revealed successful partial colonization of the donor microbiota and beneficial changes in the gut environment. Notably, there was an increase in overall bacterial diversity and an abundance of taxa such as *Bifidobacterium*, *Prevotella*, and *Desulfovibrio*, which continued to persist 8 weeks after treatment. Furthermore, a 2-year follow-up by the same team (Kang et al., 2019) showed that most GI improvements were maintained, and the improvement in ASD-related symptoms became more pronounced after treatment ended. One year later, the team reported (Kang et al., 2020) measuring comprehensive metabolites in children with ASD post-treatment, stating that FMT drove global changes in the plasma profile of multiple metabolic features, including nicotinate/nicotinamide and purine metabolism. The team of Li N. et al. (2021) conducted a clinical trial that found FMT to be effective in improving both GI and ASD symptoms without causing any serious complications. The results indicated that FMT significantly altered serum levels of neurotransmitters, promoted the colonization of donor microbiota, and shifted the bacterial community in children with ASD to resemble that of TD controls. Li Y. et al. (2024) administered oral freeze-dried FMT to 38 children with ASD, improving their GI and ASD-related symptoms, as well as sleep disorders, also showing altered composition of their gut bacterial and fungal microbiota.

As an emerging treatment method, FMT has demonstrated efficacy in recurrent CDI and shows potential for addressing neurodevelopmental disorders. Unlike traditional therapies, FMT aims to alter the gut ecosystem of ASD patients, potentially influencing neurotransmitter metabolism through the microbiota-gut-brain axis. Preliminary open-label studies have reported improvements in both gastrointestinal symptoms and core autistic behaviors, although these findings must be interpreted with caution given the absence of control groups. While generally considered minimally invasive, FMT has demonstrated a relatively acceptable short-term safety profile in controlled settings primarily for CDI; however, its safety profile in pediatric ASD populations has not been rigorously established.

However, it is crucial to maintain a balanced perspective regarding FMT for ASD. Despite encouraging findings in the current literature, the clinical application of FMT in ASD remains highly experimental and faces significant challenges. The available literature contains a small number of cases, trial sample sizes are generally modest, and all published studies in ASD are open-label and non-blinded, lacking placebo-controlled or double-blind designs which are essential to rule out placebo effects, especially for behavioral outcomes. There is a pressing need for larger, well-designed randomized controlled trials. Additionally, significant limitations and unresolved issues exist. Key concerns requiring resolution include the long-term safety and regulatory status of FMT for neuropsychiatric indications, potential risks such as the transmission of pathogens or multi-drug resistant organisms, variability in donor microbiota engraftment, and the lack of standardized protocols for ASD. Delays in donor screening add potential safety risks. Furthermore, important ethical considerations arise when applying FMT to pediatric ASD populations. These include the vulnerability of children as research subjects, the challenges of obtaining truly informed consent, and the unresolved question of whether the potential benefits justify the unknown long-term risks in a developing child. Moreover, the precise mechanisms underlying any therapeutic effect of FMT in ASD remain unclear, indicating that its scientific basis requires further exploration. Public acceptance and regulatory approval for this use are also pending. Overall, while FMT offers a novel research avenue for ASD, it must be approached with caution. Continued rigorous research is needed to elucidate potential therapeutic mechanisms, optimize treatment protocols, establish safety profiles, and develop unified operational standards and regulatory systems before clinical application can be considered.

### 5.3 Dietary interventions

In addition to emphasizing microecological regulation through probiotics and prebiotics, diet serves as a non-pharmacological and highly manageable intervention that can influence the gut-brain axis through various pathways. On one hand, diet can modulate gut microbes and their metabolites; on the other hand, it impacts brain function by influencing neural pathways (Horn et al., 2022). To date, some studies have indicated that dietary adjustments have been associated with improvements in gut health, modulation of immune-inflammatory responses, and reductions in ASD manifestations (Fang et al., 2025), which has increased attention to the influence of the gut-brain axis pathway.

It should be noted at the outset that the majority of evidence supporting dietary interventions for ASD derives from observational studies, small uncontrolled trials, or anecdotal reports, with very few high-quality randomized controlled trials available. This limitation should be kept in mind throughout the following discussion.

Based on these mechanisms, various dietary intervention strategies have been proposed and implemented for ASD. Among the specific nutrient restriction diets, Matthews JS highlighted that the gluten-free/casein-free (GFCF) diet is currently the most widely used dietary strategy for ASD, receiving the highest rating for overall symptom improvement due to its avoidance of gluten and casein-containing foods (Matthews and Adams, 2023). The expression of IgA and IgG antibodies in children with ASD can be induced to increase by gluten and casein, potentially exacerbating symptoms. Furthermore, the metabolites of gluten and casein peptides in the small intestine may contribute to behavioral problems in children with autism (Hartman and Patel, 2020). A meta-analysis by Quan et al. (2022) showed that a GFCF diet can reduce stereotyped behaviors and improve cognition in children with ASD. In the study by Matthews JS, dietary strategies represented by the ketogenic diet (KD) scored the highest for autism symptoms (Matthews and Adams, 2023). It can exert neuroprotective effects on the microglial cell line (BV2) through its primary ketone body, beta-hydroxybutyrate (BHB). This compound enhances the anti-inflammatory capacity of microglia by significantly reducing the expression of the pro-inflammatory cytokine IL-17 and increasing the levels of the anti-inflammatory cytokine IL-10, thereby limiting the inflammatory response (Polito et al., 2023). After 4 months of KD, the observation of gut microbiota in autistic children revealed a significant increase in Lactobacillales, indicating a modulation of gut microbiota composition. Additionally, Barnhill et al. (2020) found that gastrointestinal symptoms and behavioral problems improved in children with ASD following the KD intervention. As an effective high dietary fiber diet for clearing toxins produced in the gut (Mohammad et al., 2022), Increased fiber intake is associated with an increased abundance of beneficial genera such as *Lactobacillus* and *Bifidobacterium*, and may suppress the growth of opportunistic pathogens like *Desulfovibrio*. This dietary change also helps reduce systemic inflammatory responses and lower the levels of inflammatory chemokines such as IL-1β, IL-6, MCP-1, and TNF-α (Chen et al., 2023). Dietary fiber supplies essential nutrients that promote the diversity of gut microbiota. Upon fermentation, it produces SCFAs, inhibits the activity of histone deacetylase (HDAC), and influences the pro-inflammatory signaling pathway NF-κB (Gasaly et al., 2021; Zhang, 2022). Increasing dietary fiber intake can lower the gastrointestinal symptom scores in individuals with ASD (Tomova et al., 2020). The Mediterranean diet primarily consists of olive oil, fish, whole grains, fruits, vegetables, and nuts. Key components of this diet include polyphenols, polyunsaturated fatty acids (PUFA) ω-3, and fiber. The high levels of fiber and antioxidant-rich polyphenols in the diet can modulate the composition of the gut microbiota, promoting the growth of beneficial bacteria (Barber et al., 2023), having a neuroprotective effect on ASD patients (Ristori et al., 2019). In general, these dietary patterns can alleviate ASD symptoms by improving gut health and reducing inflammation levels. However, each strategy has its limitations. For instance, the GF/CF diet requires careful consideration of sensitivity, tolerance, and the risk of potential nutrient deficiencies, such as calcium. The ketogenic diet demands attention to possible growth suppression issues. The high-fiber diet necessitates avoiding digestive discomfort, while the Mediterranean diet often struggles with low adherence in children (Baspinar and Yardimci, 2020; Fuentes-Albero et al., 2024; Li Q. et al., 2021). While different strategies are appropriate for specific populations and risk profiles, there has been a notable shift from avoiding single nutrients to embracing multi-mechanism integration and precise nutritional regulation.

Dietary regimens, when compared to pharmacological interventions, are less invasive, have fewer side effects, and can be maintained long-term, making them especially suitable for children with ASD. However, clinical research in this area encounters challenges. Most existing studies are limited in sample size or are observational in design, and there is a notable absence of large-scale randomized controlled trials (Yu et al., 2022). Furthermore, the prevalence of food allergies and adverse eating habits is higher in children with autism spectrum disorder, leading to low adherence to medical diets (Coppola et al., 2024). Additionally, significant differences in genetic backgrounds, microbiota configuration, and nutritional statuses among ASD patients lead to considerable individual variation in the effectiveness of the same dietary interventions. Future research must standardize detection methods for microbiomes and inflammatory markers, thoroughly elucidate the molecular mechanisms by which different dietary components influence neurodevelopment via the gut-brain axis, and design precise nutritional intervention plans based on individual genetic and microbiota characteristics. Through multidisciplinary collaboration and multimodal strategies, dietary therapy is expected to play a larger role in the non-pharmacological treatment of ASD.

A key interpretive challenge in dietary intervention studies is distinguishing the effects of direct microbiota modulation from those of improved overall nutritional status. For example, benefits from a gluten-free/casein-free (GFCF) or Mediterranean diet may stem not only from altering microbial composition but also from reducing food intolerances, increasing micronutrient intake, or decreasing systemic inflammation. Future dietary intervention studies must include detailed nutritional assessments and mechanistic measures (e.g., metabolomics, barrier function) to dissect these contributing factors.

## 6 Current Challenges, methodological Limitations, and future directions

Despite growing evidence, this field faces significant methodological challenges and limitations in interpretation.

### 6.1 Methodological heterogeneity and limitations in current ASD microbiome research

Autism spectrum disorder microbiome research is dominated by observational and cross-sectional studies, which inherently limit causal inference. This raises the central issue of reverse causality–whether observed gut microbiota alterations contribute to ASD pathogenesis or are instead a secondary consequence of ASD-associated factors. Core features of ASD, including highly selective eating patterns and restricted diets, altered gastrointestinal motility (e.g., chronic constipation or diarrhea), elevated stress responses, and the frequent use of medications such as antibiotics and psychotropics, are all capable of independently shaping the composition and function of the gut microbiota. This ‘chicken-or-egg’ problem cannot be resolved by cross-sectional study designs. Disentangling cause from effect requires longitudinal birth cohort studies that begin in early infancy, prior to ASD diagnosis, allowing researchers to track whether early-life microbiota deviations precede and predict later neurodevelopmental outcomes. Mechanistic animal models, in which causality can be directly tested through microbiota transplantation and controlled interventions, also represent an essential complementary approach. Large heterogeneity exists across studies due to variations in participant age, geography, dietary patterns, and sampling methodologies (stool vs. mucosal biopsies). Furthermore, confounding factors like gastrointestinal comorbidities and differences in stool consistency are often not fully controlled. Substantial technical variability exists at every step, from sample collection and DNA extraction methods (e.g., 16S rRNA gene sequencing vs. shotgun metagenomics) to bioinformatic analysis pipelines, impacting the comparability and reproducibility of results. The lack of longitudinal studies starting in infancy, before ASD diagnosis, is a critical gap that limits our ability to define developmental trajectories and investigate causal relationships between early microbiota shifts and subsequent neurodevelopment.

Given these constraints, it is essential to critically appraise the strength of evidence for proposed interventions. In evaluating probiotic studies, we prioritize findings from randomized controlled trials (RCTs), note the stage of evidence from open-label/pilot studies, and emphasize the strain-specificity of effects. Similarly, as noted in preceding sections, the evidence for FMT in ASD currently rests on small-scale, open-label trials, which necessitate cautious interpretation due to potential placebo effects and the absence of blinding. To build a robust evidence base, future research must prioritize well-designed, multi-center, double-blind RCTs for all MGBA-targeted interventions.

Given the narrative and integrative scope of this review, formal quality assessment tools (e.g., Newcastle-Ottawa Scale, Cochrane Risk of Bias) were not systematically applied to each of the ∼150+ cited studies, as this approach is more characteristic of a systematic review/meta-analysis. Instead, our critical evaluation of study quality and reproducibility has been woven throughout the manuscript, with particular attention to sample sizes, study designs, blinding status, methodological heterogeneity, and potential confounding variables across the cited literature.

### 6.2 Future directions

To overcome current limitations and move the field forward, future studies should aim to:

Conduct longitudinal birth cohort studies to establish temporal and potentially causal links.

Employ multi-omics integration (metagenomics, metabolomics, metatranscriptomics) with parallel neuroimaging and clinical phenotyping to move beyond associations and unravel mechanistic pathways.

Incorporate deep phenotyping of participants to account for the immense heterogeneity within the ASD spectrum, potentially enabling microbiome-based stratification of sub-populations.

Expand beyond the bacterial microbiota to comprehensively investigate the roles of the gut virome (phageome) and mycobiome (fungal community), which are emerging as key modulators of microbial ecology and host immunity.

Leverage advances in synthetic biology and microbiome technology to develop next-generation therapeutics, such as engineered probiotics designed to deliver specific neuroactive metabolites (e.g., butyrate, GABA), defined postbiotic formulations (purified bacterial components or metabolites), and synthetic microbial consortia tailored to correct ASD-associated dysbiosis. The convergence of these fields with personalized medicine approaches may ultimately enable the development of more precisely targeted interventions.

## 7 Discussion

Building on the methodological challenges and future directions outlined in section “6 Current Challenges, methodological Limitations, and future directions,” we here provide a concise synthesis of the key translational considerations that emerge from the current evidence base. Intervention strategies based on the microbiota-gut-brain axis (MGBA) provide new pathways for the diagnosis and treatment of autism spectrum disorder (ASD), yet their clinical translation faces several challenges. The high heterogeneity among ASD patients regarding genetic backgrounds, baseline gut microbiota compositions, and environmental exposures may contribute to variable responses to interventions, making it difficult to establish universal therapies. Current clinical evidence primarily stems from short-term, small-sample trials, lacking support from large-scale, long-term follow-up data. For invasive therapies such as Fecal Microbiota Transplantation (FMT), potential long-term risks, such as microbial dysbiosis and metabolic abnormalities, require systematic evaluation. Mechanistically, the MGBA involves complex interactions among neural, immune, and metabolic pathways, and the key signaling molecules and definitive causal chains remain incompletely understood, limiting the development of targeted therapies. Furthermore, there is a lack of unified technical standards and regulatory frameworks; from probiotic strain selection and FMT donor management to dietary intervention protocols, standardization is needed to ensure reproducible efficacy and safe, controlled clinical applications. Overall, the evidence supporting MGBA-targeted interventions for ASD remains at an early stage: probiotic RCTs are few and primarily exploratory, FMT evidence is restricted to open-label case series, and dietary studies rely heavily on observational designs. These limitations must temper enthusiasm and motivate more rigorous investigation.

Future research should prioritize deepening mechanistic understanding and promoting clinical integration. The primary objective is to employ multi-omics technologies, germ-free animal models, and neural circuit manipulation techniques to accurately delineate the pathways through which specific gut microbiota and their metabolites (e.g., short-chain fatty acids) modulate the central nervous system. In clinical research, there is an urgent need for well-designed, multi-center, randomized double-blind, large-sample trials, as well as the establishment of biomarker systems correlated with clinical phenotypes to support personalized interventions. Ultimately, the success of treatment strategies will rely on the synergistic integration of microbiota-targeted interventions-such as probiotics, FMT, and dietary modulation-with existing behavioral therapies, creating a multimodal treatment framework. Through sustained interdisciplinary collaboration, MGBA research has the potential to translate basic discoveries into clinical practice, providing new solutions for ASD diagnosis and treatment.

## 8 Conclusion

This review synthesizes current evidence establishing the microbiota–gut–brain axis (MGBA) as a critical bidirectional network influencing the pathogenesis and symptomatology of autism spectrum disorder (ASD). Characteristic gut microbial dysbiosis–marked by altered abundances of Firmicutes, Bacteroidetes, and Proteobacteria, along with disruptions in short-chain fatty acid metabolism–has been linked to alterations in neural, immune, and endocrine pathways, and is thought to contribute to neuroinflammation, impaired intestinal permeability, and behavioral deficits observed in ASD. Emerging interventions targeting the gut microbiota, including specific probiotic strains, dietary approaches such as gluten-free/casein-free and ketogenic diets, and–in a highly experimental capacity–fecal microbiota transplantation (FMT), have shown promise in alleviating both gastrointestinal and core behavioral symptoms. Nevertheless, clinical translation remains constrained by the heterogeneity of ASD, variability in individual microbiome profiles, and a predominance of small-scale, non-blinded studies. Future research must prioritize mechanistic elucidation through multi-omics integration, germ-free models, and well-designed randomized controlled trials to establish causality, optimize treatment protocols, and advance toward personalized, microbiota-based therapeutics for ASD. As a narrative review, this synthesis is not intended to provide a systematic evaluation of the literature; rather, it offers a critical overview that may inform the design of future systematic investigations and hypothesis-driven clinical research.
